# The Altered Expression of *microRNA408* Influences the Arabidopsis Response to Iron Deficiency

**DOI:** 10.3389/fpls.2019.00324

**Published:** 2019-04-02

**Authors:** Àngela Carrió-Seguí, Omar Ruiz-Rivero, Laura Villamayor-Belinchón, Sergi Puig, Ana Perea-García, Lola Peñarrubia

**Affiliations:** ^1^ Departament de Bioquímica i Biologia Molecular, Estructura de Recerca Interdisciplinar en Biotecnologiaia i Biomedicina (ERI BIOTECMED), Universitat de València, Valencia, Spain; ^2^ Departamento de Biotecnología, Instituto de Agroquímica y Tecnología de Alimentos (IATA), Consejo Superior de Investigaciones Científicas (CSIC), Valencia, Spain

**Keywords:** Arabidopsis, hydrogen peroxide, iron deficiency, lignin, microRNA408

## Abstract

MicroRNAs contribute to the adaptation of plants to varying environmental conditions by affecting systemic mineral nutrient homeostasis. Copper and iron deficiencies antagonistically control the expression of *Arabidopsis thaliana microRNA408* (*miR408*), which post-transcriptionally regulates laccase-like multicopper oxidase family members *LAC3*, *LAC12*, and *LAC13*. In this work, we used *miR408* T-DNA insertion mutants (408-KO1 and 408-KO2) and a previously characterized transgenic line overexpressing *miR408* (35S:408-14) to explore how *miR408* influences copper- and iron-dependent metabolism. We observed that the altered expression of *miR408* diminished plant performance and the activation of the iron-regulated genes under iron-deficient conditions. Consistently with the low expression of the *miR408*-target laccases, we showed that the vascular bundle lignification of the *35S:408-14* plants diminished. The decrease in the phenoloxidase and ferroxidase activities exhibited by wild-type plants under iron deficiency did not occur in the 408-KO1 plants, probably due to the higher expression of laccases. Finally, we observed that the hydrogen peroxide levels under iron starvation were altered in both the *408-KO1* and *35S:408-14* lines. Taken together, these results suggest that Arabidopsis plants with modified *miR408* levels undergo multiple deregulations under iron-deficient conditions.

## Introduction

Systemic signaling between roots and shoots is required to maintain mineral nutrient homeostasis in plants cultivated under varying environmental conditions. The nutrient itself, but also other molecules such as microRNAs, transmits and signals nutritional status information for whole plant adaptation (Sunkar et al., 2007; Kehr, 2013; Zhang et al., 2016; Chien et al., 2017). In *Arabidopsis thaliana*, a group of conserved small RNAs, denoted as Cu-miRNAs, are upregulated under copper (Cu) deficiency to target nonessential cuproproteins (Abdel-Ghany and Pilon, 2008). Cu-miRNAs *miR397, miR398*, and *miR408* are highly conserved, which supports the importance of small RNA-mediated regulation in plant Cu homeostasis (Pilon, 2017). Apart from regulating local Cu deficiency responses, Cu-miRNAs also act as phloem-mediated systemic signals during Cu allocation and adaptation to metal deficiency (Buhtz et al., 2010; Kehr, 2013).

Experimental evidence indicates the interplay between Cu and iron (Fe) homeostases during different processes, including the antagonistic control of miRNA expression (Buhtz et al., 2010; Waters et al., 2012). *miR398* regulates the mRNAs of genes *CSD1* and *CSD2*, which encode cytosolic and chloroplastic Cu/Zn superoxide dismutases (Cu/ZnSODs), respectively (Yamasaki et al., 2007). Under Cu deficiency conditions, transcription factor SQUAMOSA-PROMOTER BINDING-LIKE PROTEIN 7 (SPL7) upregulates *miR398* to replace Cu/ZnSOD with the Fe superoxide dismutase (FeSOD) counterpart, probably to economize Cu for essential cuproproteins such as plastocyanin (Yamasaki et al., 2007, 2009). Conversely under Fe deficiency conditions, the downregulation of *miR398* contributes to FeSOD replacement with Cu/ZnSOD (Waters et al., 2012). Another antagonistically regulated miRNA by Cu and Fe deficiencies in phloem sap is *miR408* (Buhtz et al., 2010), but its physiological significance remains uncovered. *miR408* expression in Arabidopsis is abundant and spatially ubiquitous (Sunkar et al., 2006; Zhang and Li, 2013). *miR408* is required for proper vegetative development and is involved in the adaptation to different abiotic stresses (Zhang and Li, 2013; Zhang et al., 2014; Ma et al., 2015). *miR408* regulation under Cu deficiency is mediated by SPL7 through the binding to the GTAC motifs within the *miR408* promoter (Yamasaki et al., 2009; Bernal et al., 2012; Zhang and Li, 2013). Besides, *miR408* is also a target of HY5 (elongated Hypocotyl 5), a transcription factor that mediates responses to light (Zhang et al., 2014). *miR408* overexpression partially compensates the effects in the *spl7* and *hy5* mutants under low Cu conditions by improving the plastocyanin function (Zhang and Li, 2013; Zhang et al., 2014). Based on its role in responses to light and Cu through the HY5-SPL7 gene network, *miR408* has been proposed to act as an integrator of environmental signals in order to properly deliver Cu to plastocyanin for photosynthesis (Zhang et al., 2014). Thus a constitutive *miR408* expression improves photosynthetic performance, increases the Cu content of chloroplasts, and improves biomass and seed yield in diverse plant species (Zhang et al., 2017; Pan et al., 2018; Song et al., 2018). Cupredoxin, plantacyanin, and uclacyanin mRNAs are *miR408* targets (Abdel-Ghany and Pilon, 2008). These are blue cuproproteins that function as electron transfer shuttles between proteins (Nersissian et al., 1998; Choi and Davidson, 2011). *miR408* targets the mRNAs of the *LAC3*, *LAC12*, and *LAC13* genes encoding laccase-like multicopper oxidases (LMCOs) (LC, EC 1.10.3.2) (Abdel-Ghany and Pilon, 2008). LMCOs are extracellular glycoproteins that catalyze the oxidation of many substrates *in vitro* with simultaneous oxygen reduction (McCaig et al., 2005; Turlapati et al., 2011). Based on the wide range of substrates, it has been proposed that higher plant LMCOs could play more varied functions than initially expected (Reiss et al., 2013). Some LMCO functions could be involved in processes that affect Fe homeostasis, such as lignification, ferroxidase activity, and oxidative stress, which might account for their regulation under Fe deficiency through *miR408*.

Some LMCOs are involved in the oxidative polymerization of lignins from monolignols in secondary cell-wall formation (Berthet et al., 2011; Choi and Davidson, 2011; Zhao et al., 2013; Wang et al., 2014), and their activities are affected by *miRNA* expression (Lu et al., 2013). Cell-wall modifications through cross-linking have been suggested to affect metal chelation and mobilization (Le Gall et al., 2015; Curie and Mari, 2017). Among the potential interactions between Cu and Fe homeostases, we find metal competition for ligands during long-distance traffic under scarcity conditions (Alvarez-Fernández et al., 2014). In addition to laccases, the peroxidases that use hydrogen peroxide (H_2_O_2_) as a substrate also contribute to lignin biosynthesis *in vivo,* and H_2_O_2_ itself plays a role in cell-wall cross-linking and loosening (O’Brien et al., 2012; Kärkönen and Kuchitsu, 2015). H_2_O_2_ scavenging in the culture medium significantly decreases the amount of extracellular lignin formed in Norway spruce, and the inhibition of superoxide ($O_{2}^{-}$) synthesis, or its dismutation to H_2_O_2_ by superoxide dismutases reduces lignin content (Kärkönen et al., 2002; Karlsson et al., 2005). Rice OsLAC3 has been shown to induce H_2_O_2_ accumulation, which affects the seed setting rate and mitochondria integrity in vascular tissues and root tips (Yu et al., 2017). Given the striking similarity between OsLAC3 and L-ascorbate oxidases, a role in oxidizing ascorbate, which drives to restrain H_2_O_2_ removal, has been proposed to explain the observed phenotypes. Reactive oxygen species (ROS) could also affect Cu-Fe interactions under metal deficiency conditions (Ravet and Pilon, 2013). The delicate balance between ROS, particularly H_2_O_2_, production and scavenging during metal stress is important for diverse signaling pathways, and there is evidence for a correlation between H_2_O_2_ and plant metal tolerance (Cuypers et al., 2016).

The role proposed for certain LMCOs as putative ferroxidases would be another *miR480-*controlled process that could participate in the interplay between Cu and Fe homeostases. LMCOs participate in Fe traffic in organisms other than plants (Hoopes and Dean, 2004). Some LMCO members could be involved in redox cycles, which are necessary for the mobilization and trafficking of Fe as they contain the residues expected for this purpose (Quintanar et al., 2007; Kosman, 2010a; Turlapati et al., 2011). Among other factors, the Fe redox state depends on the ratio between ferroxidase and ferrireductase activities (Kosman, 2010b, 2018). However, experimental evidence for LMCOs being involved in metal oxidation, by acting as ferroxidases, in plants is scarce (Müller et al., 2015). All plants, except grasses, acquire Fe after the reduction of Fe^3+^ chelates by a plasma membrane ferric chelate reductase which, in *Arabidopsis,* is encoded by *FERRIC REDUCTASE 2* (*FRO2*) (Robinson et al., 1999). Upon reduction, Fe^2+^ is incorporated into the cell through a transporter encoded by IRON REGULATED TRANSPORTER 1 (IRT1) (Eide et al., 1996; Vert et al., 2002). IRT1 substrate availability depends on free external Fe (not bound to inorganic and organic complexes) and the Fe^2+^/Fe^3+^ratio, according to external redox status conditions and the enzymatic activity of ferrireductases. Helix-loop-helix type transcription factor FIT (bHLH29) is involved in Fe acquisition and remobilization (Colangelo and Guerinot, 2004). Other bHLH subgroup Ib factors (bHLH38, bHLH39, bHLH100, and bHLH101) could act in concert with FIT (Yuan et al., 2008; Wang et al., 2013). In the present work, we explore the effect of Fe deficiency on seedlings with altered *miR408* levels by analyzing *LMCO* mRNA levels, enzymatic activities, and Fe deficiency responses.

## Materials and Methods

### Plant Growth Conditions and Treatments

*Arabidopsis thaliana* ecotype *Columbia* (Col-0) was used as the control wild type (WT). The three *miR408* altered expression transgenic lines used herein, the *miR408*-overexpressing line 14 driven by the cauliflower mosaic virus 35S promoter (35S:408-14) and the two T-DNA insertion lines SALK_038860 (408-KO1) and SALK_121013.28.25.n (408-KO2), have been previously described (Zhang and Li, 2013; Ma et al., 2015). Seeds were surface-sterilized by sequential washes in 70% ethanol (5 min), bleach (5 min), and water (2 × 2 min) and were resuspended in 0.1% agar (w/v) and grown on plates containing 1/2 Murashige and Skoog (MS) medium supplemented with sucrose 1% (w/v). The 1/2 MS medium containing 1 μM CuSO_4_ and 50 μM Fe citrate was used for the metal sufficiency control conditions (50 μM Fe). Metal deficiency was obtained by home-made 1/2 MS with no added CuSO_4_ (0 μM Cu) or 5 μM Fe citrate for slight Fe deficiency (5 μM Fe) and 0 μM Fe citrate for severe Fe deficiency (0 μM Fe). For the assays of phenoloxidase and ferroxidase activities, 100 μM ferrozine, a Fe chelator, was included to provide severe deficiency growing conditions (-Fe). For severe Cu deficiency conditions, 100 μM of Cu chelator bathocuproine disulfonate (BCS) was added to the growth medium (-Cu). Intermediate photoperiodic conditions (12 h light, 20–23°C/12 h darkness, 16°C) were applied. Root length was measured by the Image J 1.42 q software.^1^

### Chlorophyll and Hydrogen Peroxide Contents and Lignin Staining

The chlorophyll-a content of the Arabidopsis seedlings was determined by the trichlorometric method (Parsons and Strickland, 1963) and estimated by the equation of Lichtenthaler (1987). Hydrogen peroxide (H_2_O_2_) was detected by the brown polymerization product, formed by a reaction with diaminobenzidine tetrahydrochloride (DAB) (Jambunathan, 2010).

Lignin staining was done using 0.1% phloroglucinol saturated with HCl (Wiesner stain) (Liljegren, 2010). Between 5 and 7 seedlings of each genotype and condition were placed inside an Eppendorf tube with 700 μl of the reagent mixture to be incubated for 5 min. Afterward, seedlings were washed with sterile water and photographed.

### Metal Content Determination

The fresh Arabidopsis material was washed once with 20 μM EDTA and three times with MilliQ H_2_O before being dried at 65°C for 2 days and digested with 65% (v/v) HNO_3_ and H_2_O_2_ 30% (v/v) at 140°C. The digested samples were then diluted with Millipore H_2_O (*Purelab Ultra*). The Cu and Fe contents were determined by mass spectrometry with inductively coupled plasma (ICP-MS Agilent technologies) at the SCSIE (Universitat de València) using the manufacturer’s standard solutions for the calibration curves.

### Gene Expression Analysis by Real-Time Quantitative PCR

The total RNA isolation, reverse transcription, and RT-qPCR analyses were performed as described in Carrió-Seguí et al. (2016). The forward (F) and reverse (R) sequences for the specific primers are shown in Table SI. To transform the fluorescent intensity measurements into relative mRNA levels, a two-fold dilution series of a mixture containing an equal amount of each cDNA sample was used, and standard curves were constructed for all the studied genes. The *UBIQUITIN10* reference gene was used for data normalization. Each sample was analyzed in biological replicates, and the mean ratios ± SD were calculated.

### Phenoloxidase and Ferroxidase Activities

Total proteins were extracted from the 7-day-old seedlings frozen in liquid nitrogen in the extraction buffer [400 mM NaCl, 2 mM MgCl_2_, 0.2% (p/v) sucrose, 20 mM Tris, PMSF 1 mM, pH 8.0, with HCl] at a ratio of 1:2 (p/v). Samples were centrifuged at 12,000 rpm for 10 min (4°C), and the supernatant was used as a crude extract. Total proteins were quantified (Bradford, 1976), and 500 μg were loaded with the nondenaturing loading buffer in the 12% SDS gels.

The phenoloxidase activity in gels was detected by using 3 mM p-phenylenediamine as the substrate (Lang et al., 2012). For the ferroxidase assay, the protocol of Hoopes and Dean (2004) was slightly modified as follows: gel was incubated for 1 h in 100 mM sodium acetate buffer, pH 5, with 5% (v/v) glycerol and 10 mM CuSO_4_, followed by a 1 h incubation period in 100 mM sodium acetate buffer, pH 5, with 0.4 mM FeSO_4_. After washing twice with distilled water and kept in the darkness at a relative humidity of 30% overnight, gel was revealed with 15 mM ferrozine. In the phenoloxidase assay, the bands around 65 kDa were quantified three times with the Image J 1.42 q software.^1^

### Statistical Analyses

The statistical differences in gene expression analyses were identified by the pair-wise fixed reallocation randomization test (*p* < 0.05) (Pfaffl et al., 2002). One-way ANOVAs were performed for the other parameters. The significant differences between means were established after the Duncan test using the Infostat Statistics software, version 2018.^2^ Data are provided as the mean values ± SD of the different biological samples indicated in the figure legends. Asterisks indicate statistical differences (*p* < 0.05) in relation to the WT value.

## Results

### Phenotypic Characterization of Plants With an Altered *miR408* Expression Under Iron Deficiency

**Figure 1:**
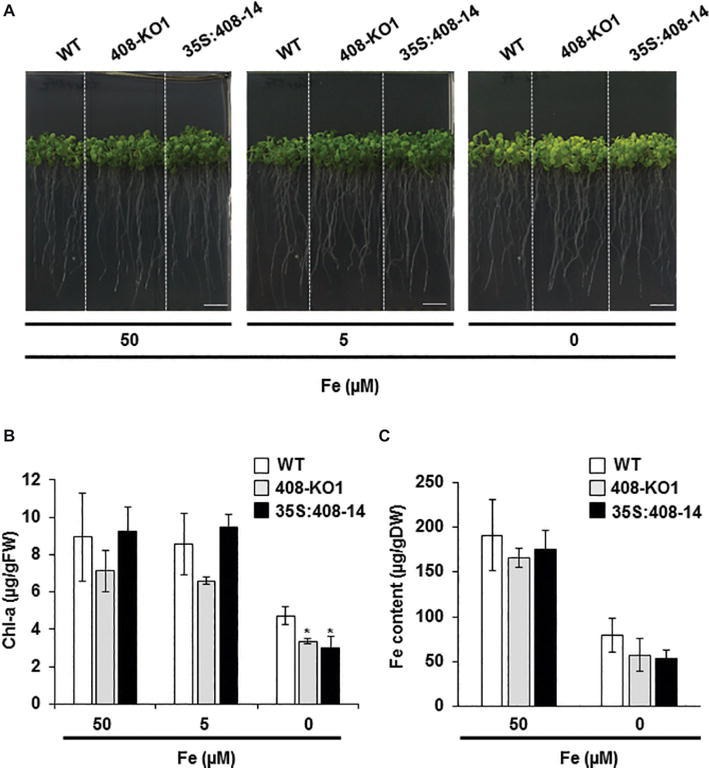
Phenotypic characterization under iron deficiency of the seedlings with altered *miR408* expression. **(A)** Photographs of representative 15-day-old seedlings grown on 1/2 MS plates containing 50, 5, and 0 μM Fe citrate. **(B)** Chlorophyll-a content and **(C)** iron content in aerial tissues measured from the wild-type (WT), 408-KO1, and 35S:408-14 seedlings grown under the same conditions as indicated in panel A. Bars represent the means ± standard deviation of three biological replicates of *n* ≥ 15 plants for each genotype. Asterisks denote statistical differences (*p* < 0.05) compared to the WT value according to the Duncan test.

The potential role of LMCOs in Fe homeostasis prompted us to conduct a physiological study of those plants with altered *miR408* expression grown under Fe deficiency. To this end, the wild-type (WT) plants, a T-DNA mutant line (408-KO1), and a transgenic line overexpressing *miR408* (35S:408-14) (Ma et al., 2015) were grown under Fe sufficiency (50 μM Fe) and under mild (5 μM Fe) and severe (0 μM Fe) Fe deficiency (Figures 1 and S1). The root length measurements indicated that only the 408-KO1 seedlings were slightly affected under the severe Fe deficiency conditions (Figure S1A). Regarding the chlorosis symptoms observed when Fe was scarce (Figure 1A; 0 μM Fe), the chlorophyll-a content of both the plants with altered *miR408* levels was significantly lower than that of the WT under severe Fe deficiency (Figure 1B; 0 μM Fe). However under Fe sufficiency and mild Fe deficiency, the chlorophyll-a content of the three lines was roughly similar (Figure 1B). To ascertain whether the altered *miR408* expression could influence metal content, Fe and Cu levels were measured in the aerial parts of the WT, 408-KO1, and 35S:408-14 lines (Figures 1C and S1B). As previously reported (Waters et al., 2012), the Cu levels in the WT seedlings were slightly higher under Fe deficiency compared to Fe sufficiency. Cu content did not change in the plants with altered *miR408* expression under Fe deprivation compared to the control under these experimental conditions (Figure S1B). The Fe content in the 408-KO1 and 35S:408-14 lines remained essentially identical to the WT plants (Figure 1C). These results indicate that proper *miR408* expression is necessary for Arabidopsis plants to adequately perform under iron-deficient conditions.

### Lignification Under Iron Deficiency Is Affected in the Plants With Altered *miR408* Expression

**Figure 2:**
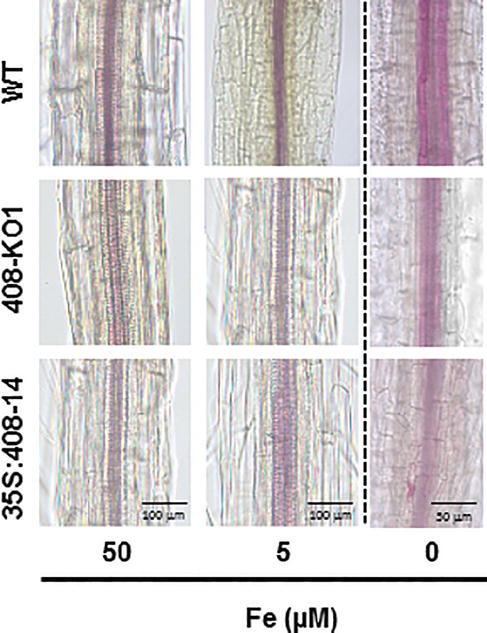
Lignin staining under iron deficiency conditions of the altered *miR408* expression seedlings. Photographs of the representative lignin stained stems from the 20-day-old seedlings from the wild-type (WT), 408-KO1, and 35S:408-14 lines grown on 1/2 MS plates containing 50, 5, and 0 μM Fe citrate, pH 5.6. Stems (*n* ≥ 10 for each genotype) were stained with phloroglucinol saturated with HCl and photographed under a microscope. The length of the bars indicates 100 μm in 50 and 5 μM Fe citrate conditions and 50 μm in 0 μM Fe citrate.

As certain LMCOs have been involved in the lignification process (Liang et al., 2006; Bonawitz and Chapple, 2010), we wondered whether there was a correlation between the degree of *LMCO* gene expression (*LAC3*, *LAC12*, and *LAC13*) and lignification. To this end, the WT, 408-KO1, and 35S:408-14 seedlings were grown under the control, slight, and severe Fe deficiency conditions (Figures 2 and S2). As previously reported (Rodríguez-Celma et al., 2016), we observed that the lignin staining of the WT vascular cylinder increased under the low Fe conditions (Figures 2 and S2). The lignification of the vascular bundles in the WT seedlings was more evident in the aerial part (Figure S2). The general reduction in lignin content observed in both the seedlings with altered *miR408* expressions under the Fe deficiency conditions was noteworthy (Figures 2 and S2).

**Figure 3:**
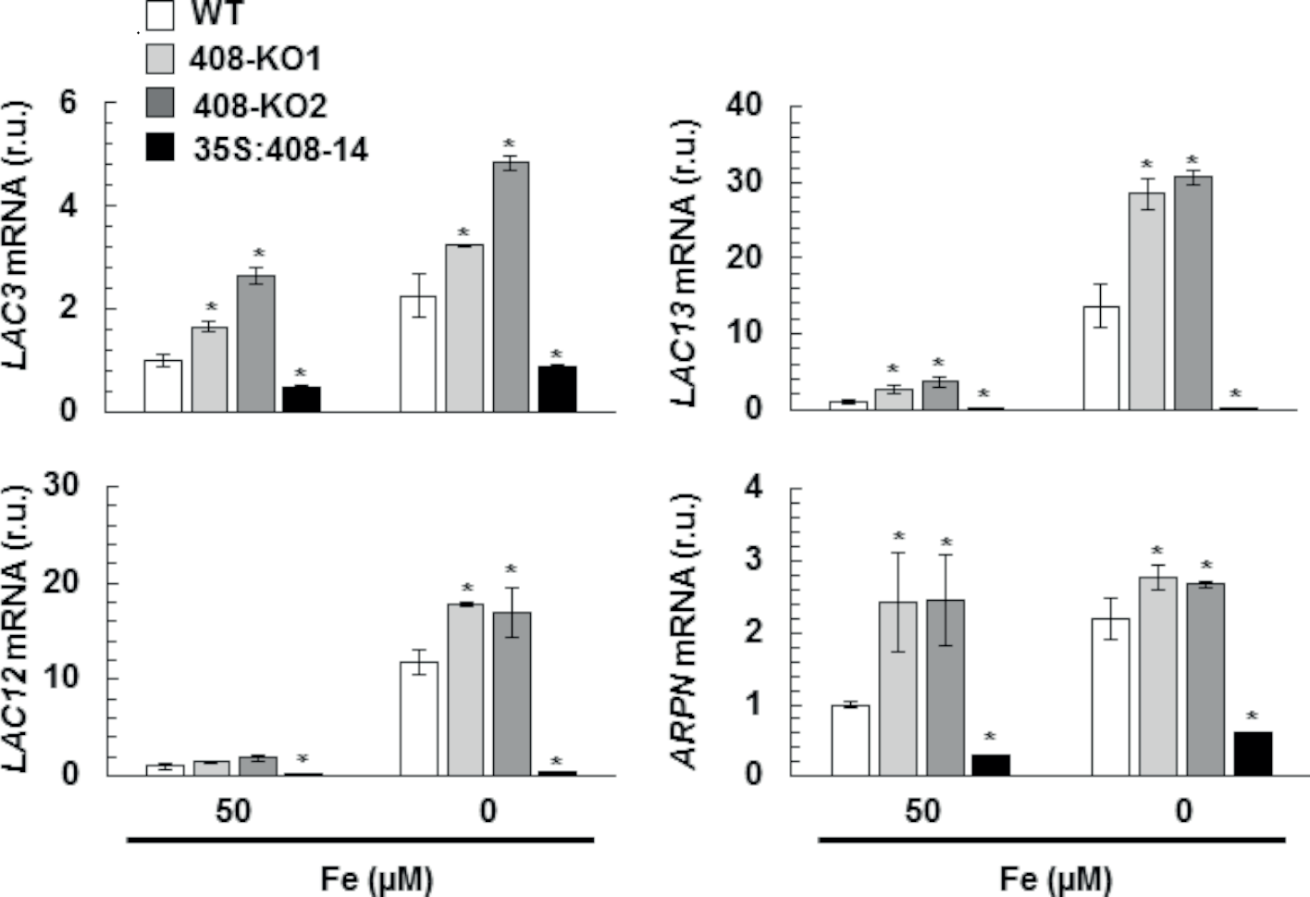
Gene expression of the *miR408* targets under iron deficiency. *LAC3, LAC12, LAC13,* and *ARPN* relative expression. The 15-day-old wild-type (WT), 408-KO1, 408-KO2, and 35S:408-14 seedlings grown in 1/2 MS medium containing 50 and 0 μM Fe citrate. Total RNA was extracted and analyzed by RT-qPCR with specific oligonucleotides for *LAC3, LAC12, LAC13*, and *ARPN.* The *UBIQUITIN10* gene was used for data normalization, and the expression is shown in relative units (r.u.). Values correspond to the arithmetic means (2^−∆∆Ct^) ± standard deviation of at least three biological replicates (*n* ≥ 25 plants). Asterisks denote significant differences for the same group of samples compared to the WT line (*p* < 0.05) based on the pair-wise fixed reallocation randomization test.

To study the *miR408* expression effects, we first corroborated that the *miR408* levels were downregulated in the 408-KO1 and were overexpressed in the 35S:408-14 lines under our experimental conditions (Figure S3A). Previous studies had shown that *miR408* is oppositely regulated by Fe and Cu deficiencies in phloem sap (Buhtz et al., 2010). In line with these data, *miR408* expression increased in the WT seedlings under Cu deficiency, but decreased under the Fe deficiency conditions (Figure S3B). The expression of *miR408* target transcript *LAC3* was checked in the WT and *miR408* mutant seedlings, with a second 408-KO line (408-KO2), under the control (1 μM Cu) and Cu-deficient conditions (0 μM Cu) (Figure S3C). As expected, the *LAC3* mRNA levels lowered in response to Cu limitation in the WT seedlings. Moreover, its levels lowered in the *miR408* overexpressing plants, but increased in the 408-KO mutants (Figure S3C), which indicates that the *LAC3* target expression responded to both the *miR408* levels and Cu deficiency in accordance with *miR408* being upregulated. To address the target responses to Fe deficiency, the WT and mutant *miR408* seedlings were grown under the control (50 μM Fe) and Fe deficiency (0 μM Fe) conditions, and the mRNA expression of *miR408* targets *LAC3*, *LAC12*, *LAC13*, and plantacyanin (*ARPN*), encoding a cyanin (Yamasaki et al., 2007), was checked by RT-qPCR (Figure 3). In agreement with *miR408* being downregulated, all the studied *miR408* targets were upregulated by Fe deficiency in the WT plants (Figure 3). Moreover, the *miR408* targets were upregulated in the 408-KO mutants and downregulated in the 35S:408-14 line under the metal sufficiency conditions, and the same results were also observed for Fe starvation (Figure 3). These results indicate that the *miR408* mRNA targets under study are properly regulated by Fe limitation and respond accordingly to the altered *miR408* expression under both the control and metal deficiency conditions (Figures 3 and S3C).

**Figure 4:**
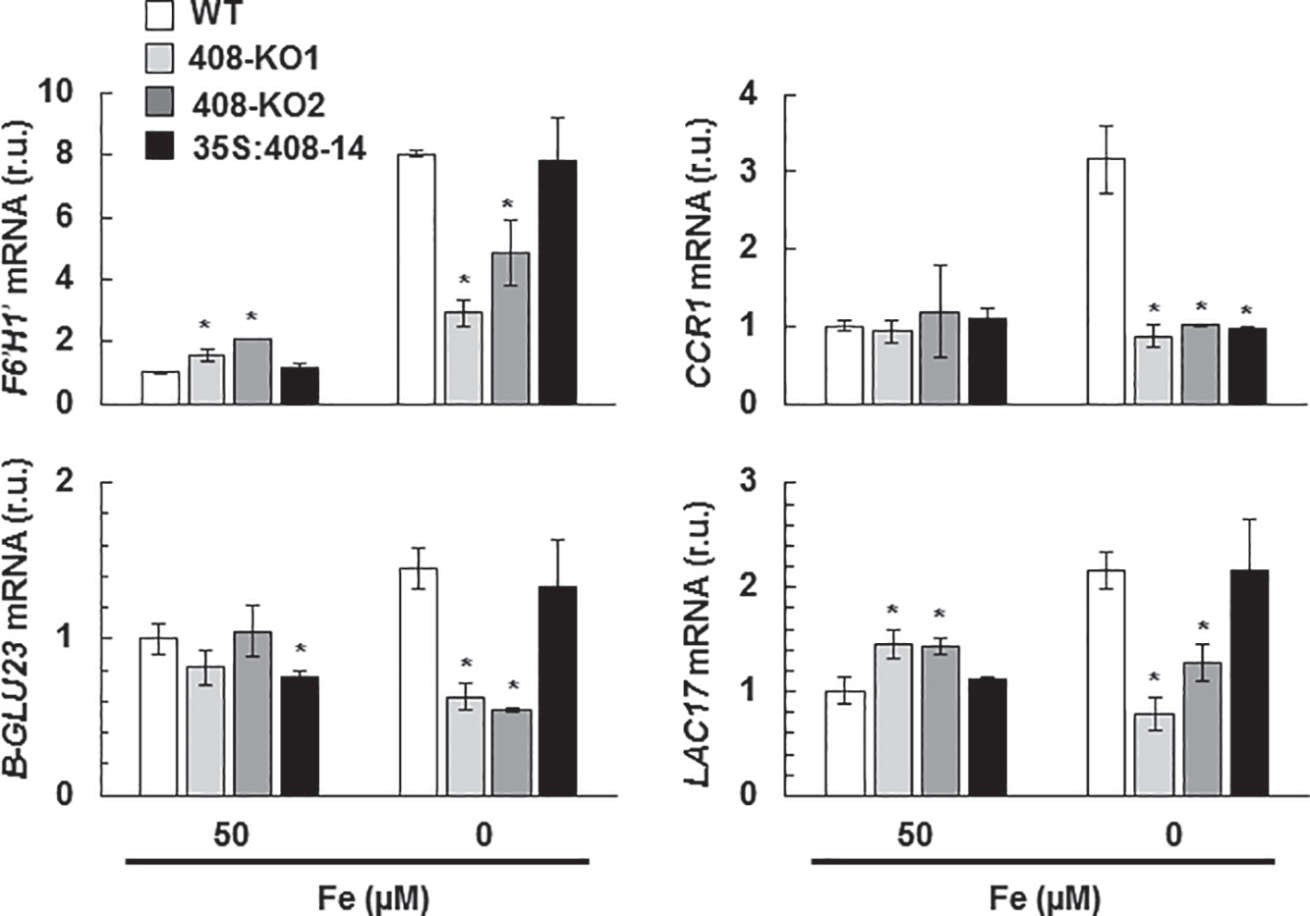
Gene expression of the lignification-related genes under iron deficiency in the seedlings with altered *miR408* expression. *F6′H1′, B-GLU23, LAC17*, and *CCR1* relative expression. The 15-day-old wild-type (WT), 408-KO1, 408-KO2, and 35S:408-14 seedlings grown in 1/2 MS medium containing 50 and 0 μM Fe citrate. Total RNA was extracted and analyzed by RT-qPCR with specific oligonucleotides for *F6′H1′, B-GLU23, LAC17*, and *CCR1.* The *UBIQUITIN10* gene was used for data normalization, and the expression is shown in relative units (r.u.). Values correspond to the arithmetic means (2^−∆∆Ct^) ± standard deviation of at least three biological replicates (*n* ≥ 3). Asterisks depict significant differences for the same group of samples *versus* the WT line (*p* < 0.05) based on the pair-wise fixed reallocation randomization test.

We analyzed whether the expression of the specific molecular markers involved in the lignin biosynthetic process (*F6’H1´*, β-*GLU23*, *CCR1*, and *LAC17;* Bonawitz and Chapple, 2010) were affected in the plants with the altered *miR408* expression grown under Fe deficiency (Figure 4). Under the Fe-limited conditions, the expression of the studied lignification-related genes significantly reduced in the 408-KO lines compared to the WT plants (Figure 4). However in the 35S:408-14 line, the mRNA levels of the lignification-related genes increased under low Fe to reach the same levels observed in the WT, except for *CCR1*, which was not induced (Figure 4). As these genes are not direct *miR408* targets, their downregulation in the 408-KO mutants is probably due to an indirect effect. With the 35S:408-14 line, the mRNA levels of the lignification-related genes increased when Fe was low, except for *CCR1,* which encodes a cinnamoyl-CoA reductase (Figure 4). These results show that *miR408* influences the expression of the genes implicated in lignin biosynthesis.

### Iron Deficiency Responses Are Affected in Altered *miR408* Expression Plants

**Figure 5:**
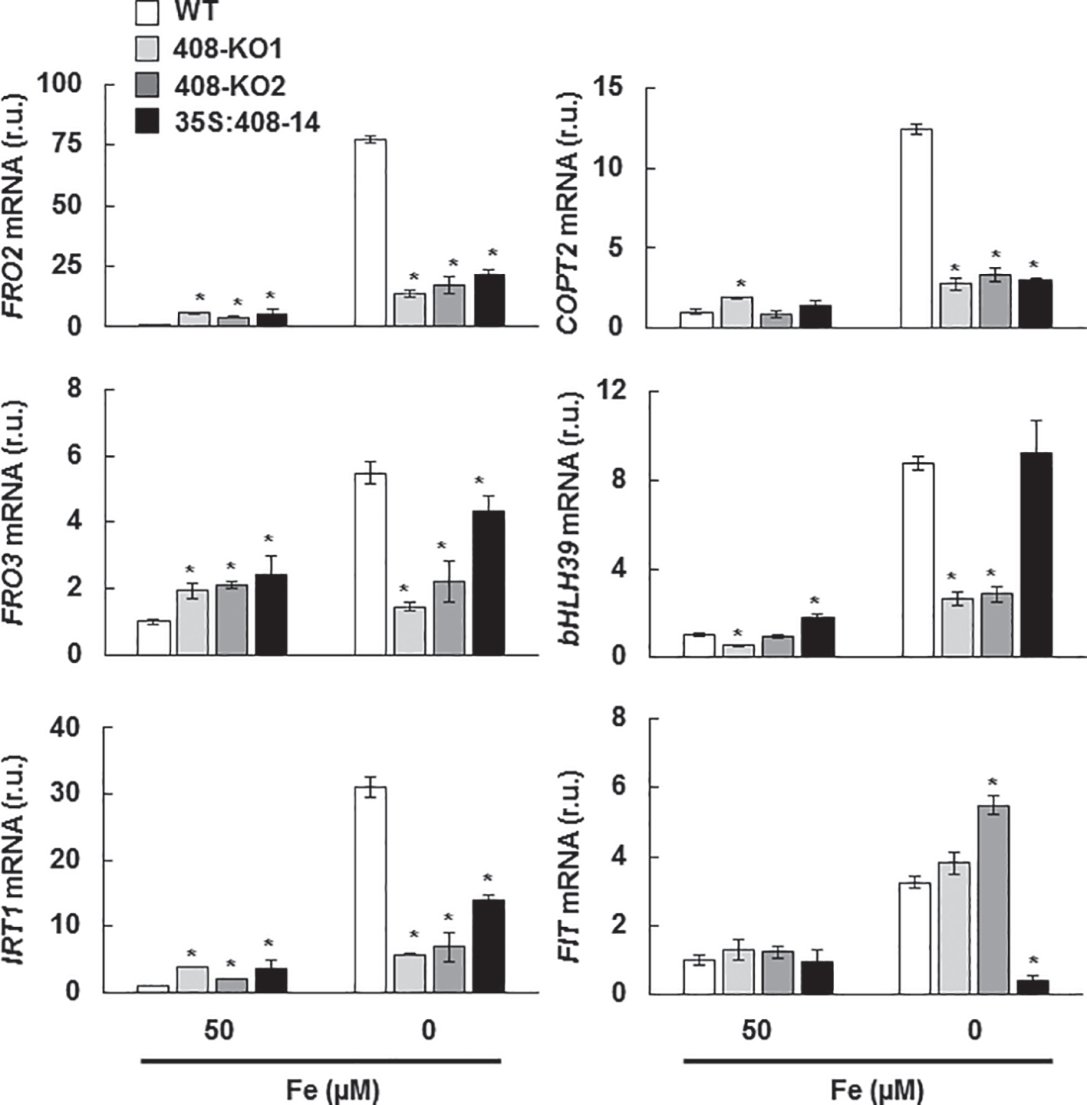
Expression of the metal homeostasis-related genes under iron deficiency in the seedlings with altered *miR408* expression. *FRO2, FRO3, IRT1, COPT2, bHLH39*, and *FIT1* relative expression. The 15-day-old wild-type (WT), 408-KO1, 408-KO2, and 35S:408-14 seedlings grown in 1/2 MS medium containing 50 and 0 μM Fe citrate. Total RNA was extracted and analyzed by RT-qPCR with specific oligonucleotides for *FRO2, FRO3, IRT1, COPT2, bHLH39*, and *FIT1.* The *UBIQUITIN10* gene was used for data normalization and the expression is shown in relative units (r.u.). Values correspond to the arithmetic means (2^−∆∆Ct^) ± standard deviation of at least three biological replicates (*n* ≥ 25). Asterisks indicate significant differences for the same group of samples *versus* the WT line (*p* < 0.05) based on the pair-wise fixed reallocation randomization test.

To determine how the plants with the altered *miR408* expression perceived Fe status, the expression of a group of molecular Fe deficiency markers was analyzed under the Fe sufficiency and deficiency conditions. The genes involved in Fe remobilization and incorporation, such as *FRO2*, *FRO3*, and *IRT1*, and Fe-regulated Cu transporter *COPT2* (Perea-García et al., 2013) were induced under Fe limitation in the WT plants (Figure 5). Unexpectedly, the expression of these Fe deficiency markers lowered in the plants with the altered *miR408* expression grown under Fe scarcity (Figure 5). In order to check the regulation of the Fe deficiency responses, the expression of transcriptional activators *bHLH39* and *FIT* (Colangelo and Guerinot, 2004; Yuan et al., 2008; Wang et al., 2013) was also analyzed (Figure 5). A differential response was observed in the 408-KO and 35S-408-14 lines. Whereas *bHLH39* expression decreased by three times in the 408-KO lines, an 8-fold reduction in *FIT* expression took place in the 35S-408-14 line compared to the WT under Fe deprivation (Figure 5).

### Characterization of Phenoloxidase and Ferroxidase Activities in Plants With Altered *miR408* Expression

**Figure 6:**
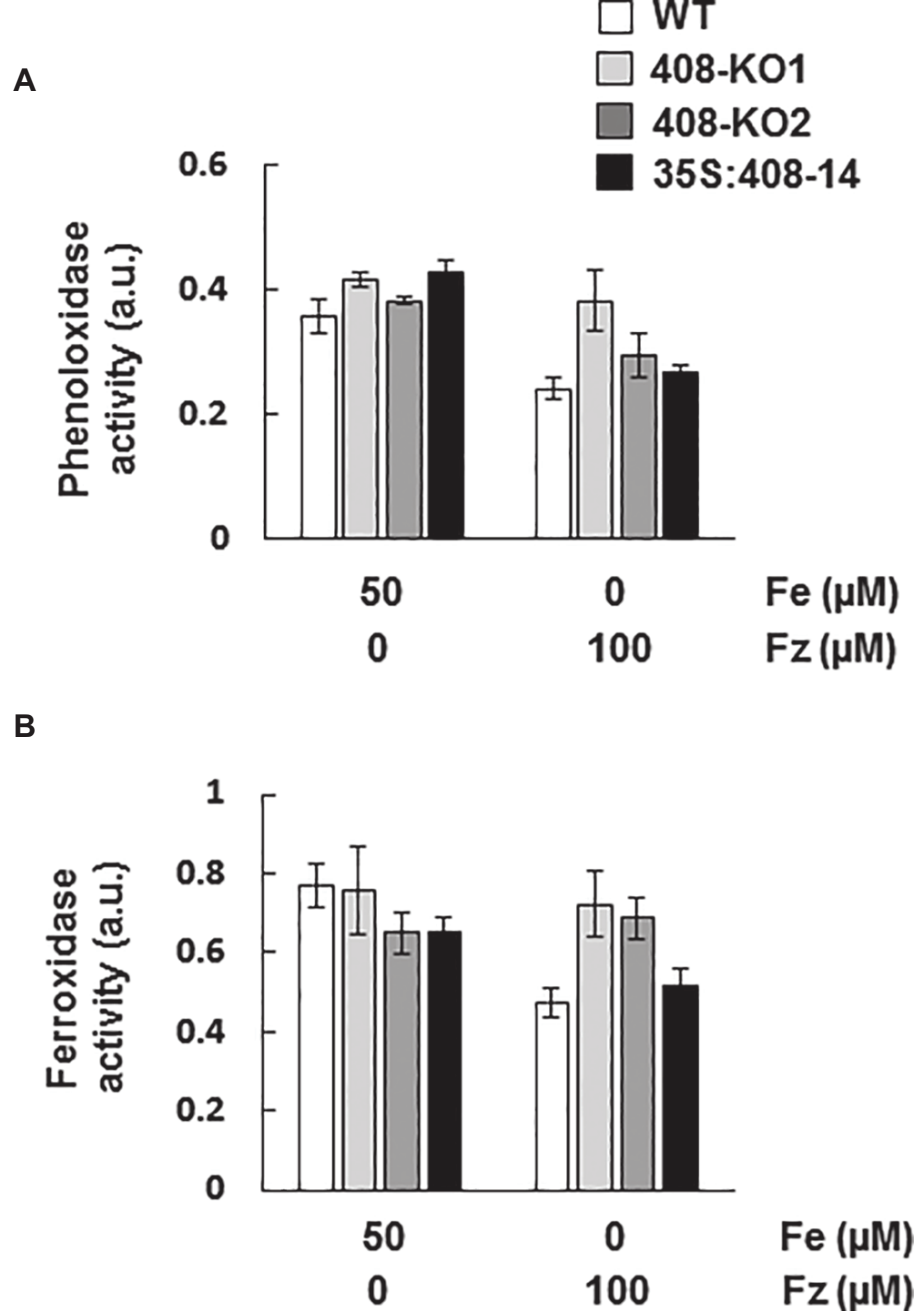
Phenoloxidase and ferroxidase activities in the seedlings with altered *miR408* expression. **(A)** Phenoloxidase and **(B)** ferroxidase activities in the gels from the 7-day-old wild-type (WT), 408-KO1, 408-KO2, and 35S:408-14 seedlings grown on 1/2 MS plates containing 50 μM Fe citrate and 100 mM ferrozine. About 100 μg of total protein extraction were used for each sample. The relative intensities of the bands corresponding to the LMCO expected MW are shown as arbitrary units (a.u.). Values are the means ± SD of three technical replicates. A representative quantification of at least three independent experiments is shown.

The wide range of substrates that LMCOs can oxidize suggests that they could be involved in many other processes apart from lignification (Reiss et al., 2013). Therefore, we decided to determine phenoloxidase activity in gel in the WT and *miR408* mutant seedlings grown under the control and Fe deficiency conditions (Lang et al., 2012; Figures 6A and S4). Remarkably, the phenoloxidase activity of the WT plants decreased under Fe deficiency (Figure 6A), despite us having shown that the *miR408*-dependent *LMCO* expression increased under these growth conditions (Figure 3). Phenoloxidase activity was slightly higher in mutants 408-KO than in the WT under Fe starvation (Figure 6A).

All laccases exhibit the characteristic conserved Cu-binding domains of LMCOs. Apart from the different Cu-binding domains formed by histidine residues, *Saccharomyces cerevisiae* ferroxidase Fet3 (Askwith et al., 1994) possesses an essential aspartic residue (D409) within its ferroxidase domain (Quintanar et al., 2007). The alignment of the *A. thaliana* laccase sequences and yeast Fet3 showed that LAC3, LAC4, LAC5, LAC10, LAC11, LAC12, LAC13, and LAC17 were LMCOs which conserved the essential ferroxidase aspartic residue (Figure S5). Among the different Cu-miRNAs with *LMCO* mRNAs as targets (Abdel-Ghany and Pilon, 2008), *miR408* is the only Cu-microRNA to target LMCO mRNAs, which encode proteins containing an aspartic residue potentially involved in Fe-binding. Thus, we hypothesized that *miR408* expression could affect ferroxidase activity. To study this possibility, ferroxidase activity was checked in gels (Hoopes and Dean, 2004) in the same samples used to measure phenoloxidase activity (Figures 6B and S4). Similar to phenoloxidase, ferroxidase activity reduced under Fe deficiency in the WT plants (Figure 6B), despite the increase in the *miR408*-dependent *LMCO* mRNAs (Figure 3). It was noteworthy that ferroxidase activity did not decrease in mutants 408-KO (Figure 6B), probably due to the expression of laccases being higher than in the WT plants (Figure 3).

### Hydrogen Peroxide Levels in Plants With Altered *miR408* Expression

**Figure 7:**
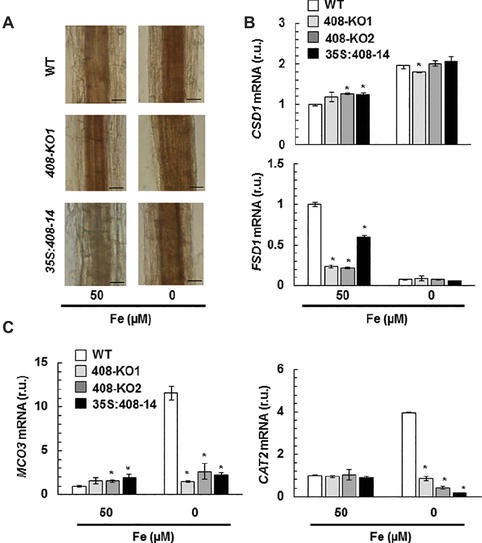
Hydrogen peroxide detection in the plants with altered *miR408* expression. **(A)** Hydrogen peroxide detected in the roots of the 15-day-old wild-type (WT), 408-KO1, and 35S:408-14 seedlings grown in 50 and 5 μM Fe citrate media. Photographs of the representative roots, where a brown polymerization product was formed by the reaction of hydrogen peroxide with diaminobenzidine tetrahydrochloride. *n* = 15 plants were stained for each genotype and condition. The length of the bars indicates 100 μm in 50 and 5 μM Fe citrate. **(B)** *FSD1* and *CSD1* **(C)** *CAT2* and *MCO3* relative expression. The 7-day-old wild-type (WT), 408-KO1, 408-KO2, and 35S:408-14 seedlings grown in 1/2 MS medium containing 50 and 0 μM Fe citrate. Total RNA was extracted and analyzed by RT-qPCR with specific oligonucleotides for *FSD1*, *CSD1*, *CAT2*, and *MCO3.* The *UBIQUITIN10* gene was used for data normalization, and the expression is shown in relative units (r.u.). Values correspond to the arithmetic means (2^−∆∆Ct^) ± standard deviation of at least three biological replicates (*n* ≥ 25 plants). Asterisks indicate significant differences for the same group of samples *versus* the WT line (*p* < 0.05) based on the pair-wise fixed reallocation randomization test.

Previous data have shown that hydrogen peroxide (H_2_O_2_) increases in the vascular bundles of WT plants under Fe deficiency (Alvarez-Fernández et al., 2014). Thus, we decided to study H_2_O_2_ levels by diaminobenzidine tetrahydrochloride (DAB) staining in the 15-day-old seedling roots from the WT and *miR408* mutants grown under the control and slight Fe deficiency conditions (Figure 7A). Under the control conditions, high H_2_O_2_ levels were observed in the vascular bundles of mutant 408-KO1, whereas the 35S:408-14 line displayed low levels *versus* the WT plants. Moreover, when Fe was scarce, the H_2_O_2_ levels increased in all the genotypes and were higher in mutant 408-KO1 and lower in the 35S:408-14 line compared to the WT line (Figure 7A). Therefore, we checked whether the expression of a set of oxidative stress genes involved in H_2_O_2_ metabolism was affected under Fe deprivation in the plants with altered *miR408* expression (Figures 7B,C). We tested the expression of genes related either to H_2_O_2_ synthesis, such as *SODs* (*CSD1, FSD1*), or elimination, such as ascorbate oxidase *MCO3* and catalase *CAT2* (Cuypers et al., 2016). We observed that *FSD1* expression significantly reduced in mutants *miR408* compared to the WT plants under Fe sufficiency (Figure 7B). However under Fe deficiency, the most relevant result was the low expression of *MCO3* and *CAT2* compared to the WT in the plants with altered *miR408* expression (Figure 7C). These data reveal that *miR408* expression determines H_2_O_2_ levels and the expression of the genes related to oxidative stress.

## Discussion

Previous work has shown the antagonistic effects of Cu and Fe deficiencies on Arabidopsis *miR408* expression (Buhtz et al., 2010). In response to Cu starvation, SPL7 binds to Cu-responsive elements within the *miR408* promoter and activates its expression (Yamasaki et al., 2009). As all *miR408* targets are mRNAs that encode apoplastic cuproproteins, a role in Cu redistribution has been postulated for *miR408* under Cu depletion (Abdel-Ghany and Pilon, 2008; Zhang et al., 2014). The mRNAs from the internal cuproproteins showed enhanced expression when *miR408* was overexpressed and its targets putatively reduced (Ma et al., 2015). From this viewpoint, an apparently pernicious effect could take place under Fe deficiency because *miR408* downregulation would increase the extracellular Cu quota *versus* the internal Cu, which could harm internal metalloprotein substitutions such as the FeSOD replacement for its Cu counterpart (Abdel-Ghany and Pilon, 2008). We ruled out a putative role of *miR408* expression in metalloprotein substitution because no significant changes in *SODs* expression were observed under Fe deficiency in the plants with altered *miR408* expression (Figure 7B).

**Figure 8:**
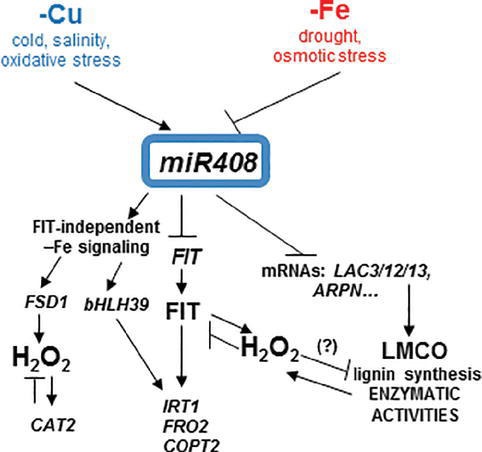
Model for the copper and iron homeostasis interplays through *miR408* regulation. Schematic representation of the main *miR408* effects on the Fe-deficiency responses observed in the present work. *miR408* directly affects the expression of the target genes, such as the mRNA from LMCOs *LAC3*, *LAC12*, *LAC13* (*LAC3/12/13*), and cyanins (i.e., *ARPN*) and their putative promiscuous LMCO role in lignin synthesis and enzymatic activities, such as phenoloxidase, ferroxidase, and ascorbate oxidase. In addition to other abiotic stresses, Cu and Fe deficiencies had the opposite effects on *miR408* regulation and their subsequent enzymatic activities. The FIT-independent and FIT-dependent signaling pathways under Fe deficiency were induced or repressed by *miR408*, respectively. Fe deficiency induced oxidative stress and hydrogen peroxide (H_2_O_2_) could play a role in the observed *miR408* effects. H_2_O_2_ could affect LMCO enzymatic activities, and at least part of the *miR408* function could be able to counteract this effect under Fe deficiency.

The signaling pathway that drives to *miR408* Fe deficiency repression and its physiological relevance remain unknown. *miR408* is both induced and repressed when faced with different abiotic stresses (Ma et al., 2015). Indeed signaling during Cu starvation would display similarities to other stresses that induce *miR408* expression, such as cold, salinity, and oxidative stress, whereas Fe deficiency signaling could converge with the stress signals that inhibit *miR408* expression, such as drought and osmotic stress (Ma et al., 2015). Based on both the physiological and molecular effects that plants with altered *miR408* expression showed under Fe limitation, the results offered herein could explain the antagonistic Fe and Cu regulation on *miR408* expression (Figure 8). Both the 35S:408-14 overexpressing line and mutants 408-KO exhibited reduced chlorophyll-a content under low Fe conditions (Figure 1). According to previous results (Ma et al., 2015), 35S:408-14 plants are resistant to the type of stresses that induce *miR408* expression and are sensitive to the stresses that inhibit its expression, among which we can now include Fe deficiency. However, whereas Fe sensitivity was observed in the 35S:408-14 seedlings, adult plants produced more seeds than the WT plants under Fe starvation (results not shown). Different possibilities were explored to understand these phenotypes. First, the role of the *miR408 LMCO* mRNA targets in the lignification process, which could affect metal translocation to the aerial part and shoot-to-root Fe deficiency signaling (Le Gall et al., 2015; Curie and Mari, 2017). Second, the potential function of LMCOs as ferroxidases and their influence on both Fe incorporation and mobilization. Finally, a putative role of LMCOs in oxidative stress and, more specifically, in H_2_O_2_ generation could affect metal transport.

Regarding the lignification process, some laccases, such as LAC4, LAC15, and LAC17, are directly involved in lignin synthesis in Arabidopsis (Liang et al., 2006; Berthet et al., 2011). Lignification is affected by *miR857* and *miR397* in both Arabidopsis and trees, such as Populus, where miRNAs act as negative regulators of laccase expression and are involved in the control of the secondary growth of vascular tissues (Lu et al., 2013; Wang et al., 2014; Zhao et al., 2015). Long-distance regulatory circuits have been identified for Fe deficiency signaling. Fe homeostasis is controlled at local and systemic levels by a wide range of signaling molecules, including ions, hormones, and metabolites (Giehl et al., 2009). Fe deficiency promotes increased lignification (Alvarez-Fernández et al., 2014). If laccases LAC3, LAC12, and LAC13 were involved in lignification, the repression of *miR408* by Fe deprivation (Figure S3B), and the subsequent increase in *LAC3*, *LAC12* and *LAC13* expression (Figure 3), if redounded in increased protein activity, would lead to enhanced lignification under Fe deficiency, as was indeed observed (Figure 2). By applying the same reasoning, lignification should be higher in 408-KO mutants and lower in the 35S:408-14 line as *LAC3*, *LAC12*, and *LAC13* expression was up- and downregulated, respectively (Figure 3). Instead, lignification under Fe deficiency was impaired in both 408-KO mutants and the 35S:408-14 line (Figure 2). However, we cannot rule out a direct role of the *miR408* target *LMCOs* in the lignification process, and it is possible that their upregulated expression would cause reduced Cu availability for other LMCOs, including those involved in lignin biosynthesis. The inhibited expression of the key regulatory genes in the lignin pathway in both 408-KO mutants and the 35S:408-14 line (Figure 4) could account for the reduction observed in lignin in these lines.

Although both the 35S:408-14 line and the 408-KO mutants showed slightly increased responses in the expression of the Fe deficiency markers under Fe sufficiency, as well as a general defect under Fe deficiency, the molecular reasons behind these effects differed in both plant types. A reduced *FIT* expression was observed in the 35S:408-14 line under low Fe conditions, which suggests that low *miR408* levels could be required for *FIT* expression and the subsequent Fe deficiency responses. Accordingly, FIT-independent Fe deficiency responses have been described for limited Cu, when *miR408* expression is induced (Waters et al., 2012). On the contrary, 408-KO mutants displayed problems in *bHLH39* expression, which could also account for their defective Fe deficiency responses. Different processes that are independent of FIT affect the expression of the Ib subgroup *bHLH* genes, including *bHLH39*. IVc subgroup members of the bHLH transcription factor family bind to the promoters of the Ib subgroup *bHLH* genes, and positively regulate Fe deficiency responses (Zhang et al., 2015). The dimethylation of histone H4R3 in the chromatin of the Ib subgroup *bHLH* genes is involved in Fe deficiency responses (Fan et al., 2014). Taken together, distinctive circumstances that depend on FIT or bHLH39 could be responsive of the defective Fe deficiency signaling in plants with high and low *miR408* expression levels, respectively.

A significant decrease in both phenoloxidase and ferroxidase activities was observed under Fe deficiency in the WT plants (Figure 6), but its cause remains unsolved. These activities also reduced in the *spl7* mutant, which exhibits exacerbated defects in root-to-shoot Fe translocation under low Cu conditions, but no responsive enzymes were identified (Bernal et al., 2012). In our study, Fe deficiency downregulated *miR408* and led to the increase in *miR408* target *LMCO* expression (Figure 3), which suggests a compensatory effect to counteract the negative effect of Fe scarcity on enzymatic activities. Thus, no decrease in phenoloxidase and ferroxidase activities was observed in mutants 408-KO at higher levels of *miR408* target *LMCOs*. This result could indicate that both activities were affected by the *miR408* expression levels, which would evidence a rather promiscuous range of activity for LMCOs, as previously suggested (Reiss et al., 2013).

LOW PHOSPHATE RESPONSE1 (LPR1), a cell wall-targeted ferroxidase, is involved in root growth inhibition when phosphate deficiency occurs by triggering Fe-stimulated apoplastic ROS generation and cell wall modifications, which impair cell-to-cell communication and meristem maintenance (Müller et al., 2015). Our results matched the concomitant increase in ferroxidase activity and ROS generation in mutant 408-KO under Fe scarcity conditions compared to the WT (Figures 6B, 7A). The results shown herein also agree with the proposed role of LMCOs functioning as ascorbate oxidases and, subsequently, with increasing H_2_O_2_ levels by competing with ascorbate peroxidases for reduced ascorbate (Yu et al., 2017). Hence according to the levels of the *miR408* target *LMCOs* (Figures 3, 7A), H_2_O_2_ levels increased in mutant 408-KO and decreased in the 35S:408-14 line compared to the WT. Moreover, the fact that H_2_O_2_ levels increased in all cases under low Fe conditions (Figure 7A), which agrees with a higher *LMCOs* expression (Figure 3), could explain the previous studies in which Fe deficiency produced enhanced root H_2_O_2_ concentrations (Alvarez-Fernández et al., 2014). However, a role for other LMCOs, such as *MCO3,* that function as ascorbate oxidases (Yamamoto et al., 2005) cannot be ruled out because *MCO3* expression was enhanced under Fe deficiency, but its expression considerably reduced in the plants with altered *miR408* levels (Figure 7C).

Redox signaling has been shown to mediate *microRNA* expression (Sunkar et al., 2012; Jagadeeswaran et al., 2014). The results obtained with antioxidant activities in mutants 408-KO (Figures 7B,C) suggest that H_2_O_2_ accumulation under Fe deprivation could be due to the poor capacity to detoxify it (Jelali et al., 2014). H_2_O_2_ content was enhanced in an FIT-dependent manner under low Fe conditions, and the FIT protein was stabilized by H_2_O_2_ in the presence of zinc-finger transcription factor ZAT12, which demonstrates that H_2_O_2_ serves as a signal for Fe deficiency responses (Le et al., 2016). If the presence of FIT is a prerequisite for H_2_O_2_ accumulation in Fe-deficient roots, the difficulties shown by the 35S:408-14 overexpressing line to express *FIT* (Figure 5) would agree with the drop in H_2_O_2_ (Figure 7A). In addition, the fact that H_2_O_2_ participates in lignification would make lignin biosynthesis in these plants even more difficult (Figures 2 and S2).

One potential explanation to justify the reduced LMCO activities observed under Fe limitation comes from previous data, indicating that H_2_O_2_ inhibits the ferroxidase activity of the major human plasma multicopper oxidase ceruloplasmin (Shukla et al., 2006; Olivieri et al., 2011; Barbariga et al., 2015). Ceruloplasmin oxidation induces the structural changes that release Cu atoms from multicopper oxidase sites, which further increases oxidative stress (Shukla et al., 2006). Subsequently, reduced extracellular ferroxidase activity redounded in intracellular Fe retention (Olivieri et al., 2011). According to these data, it is tempting to speculate that increased oxidative stress under low Fe could also inactivate ferroxidase in Arabidopsis. If this were the case, plants would face a conflict under Fe deficiency as inhibited ferroxidase activity would make Fe mobilization difficult, while a subsequent Cu increase would further compete with scarce Fe for long-distance transport. In this scenario, the downregulation of *miR408* and the subsequent increase in *LMCOs* expression could partially compensate for the negative effects of Fe depletion on ferroxidase activity. If *miR408* mediates a process whose aim is to counteract the post-translational Fe deficiency effects on LMCO activities, the opposite *miR408* regulation under Cu and Fe deficiencies could be justified.

In summary, these results suggest that Fe deficiency responses include an increase in oxidative stress, which comes in the form of H_2_O_2_, drives to enhanced lignification and affects shoot-to-root Fe signaling responses. In addition to their role in lignin biosynthesis, *miR408* and probably other Cu-miRNAs that target *LMCO* mRNAs with putative promiscuous activities, such as ferroxidases and ascorbate oxidases, would interfere with Fe deficiency responses in a complex manner.

## Data Availability

All datasets generated for this study are included in the manuscript and/or the Supplementary Files.

## Author Contributions

LP and SP conceived the idea and wrote the manuscript. ÀC-S, LV-B, and OR-R performed the physiological and molecular experiments in the mutant plants. AP-G processed the data and helped to write the manuscript.

### Conflict of Interest Statement

The authors declare that the research was conducted in the absence of any commercial or financial relationships that could be construed as a potential conflict of interest.

## Footnotes

^1^https://imagej.nih.gov/ij/

^2^http://www.infostat.com.ar
