# Supplementary material for: The Altered Expression of microRNA408 Influences the Arabidopsis Response to Iron Deficiency
Source: Front Plant Sci. 2019 Apr 2;10:324. doi: 10.3389/fpls.2019.00324 (PMC6454987; doi:10.3389/fpls.2019.00324)
Supplement: Supplementary file 1 [file Data_Sheet_1.PDF]

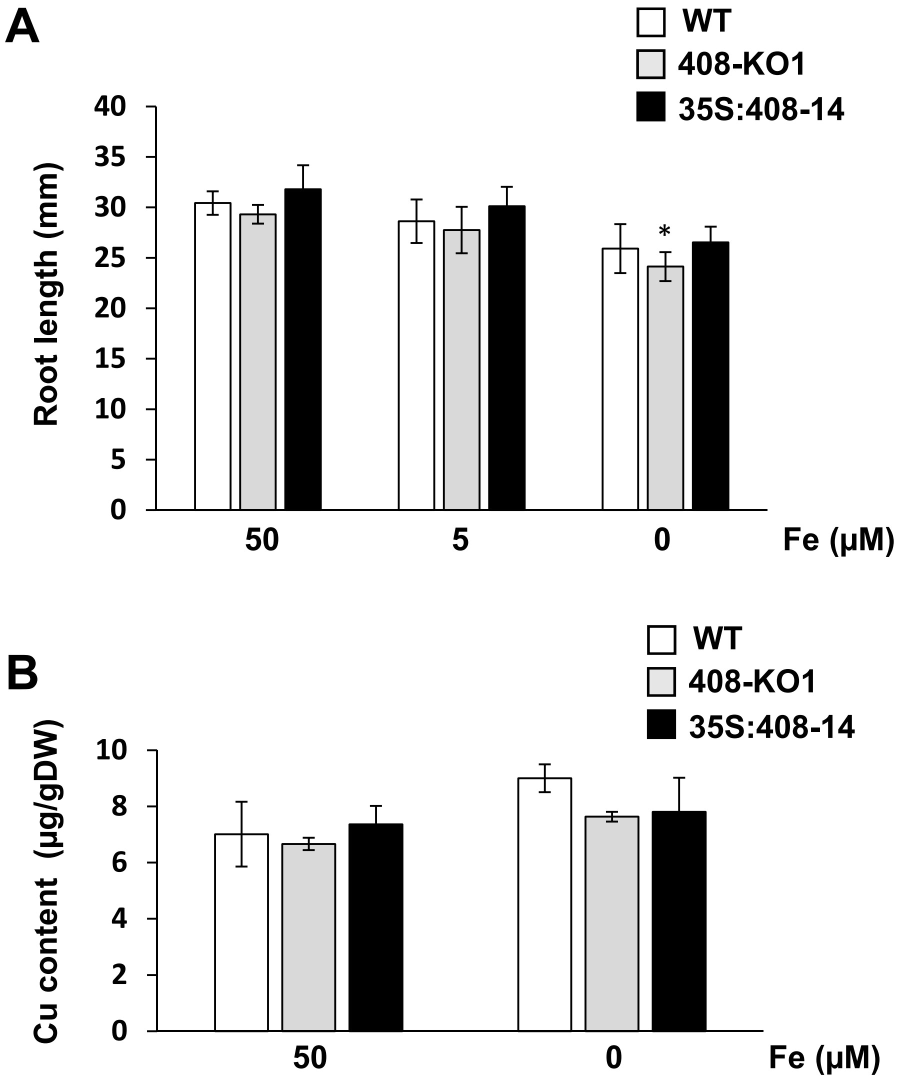

Figure S1. Phenotypic characterization of the plants with altered *miR408* expression grown under iron deficiency. (A) Root length and **(B)** copper content in the aerial tissue measured from wild-type (WT), 408-KO1 and 35S:408-14 from the 15-day-old seedlings grown under the same conditions as in Figure 1. Bars correspond to means ± standard deviation of 10 (A) and three (B) biological replicates. Asterisks indicate statistical differences ($P < 0.05$) to the WT value according to the Duncan test.

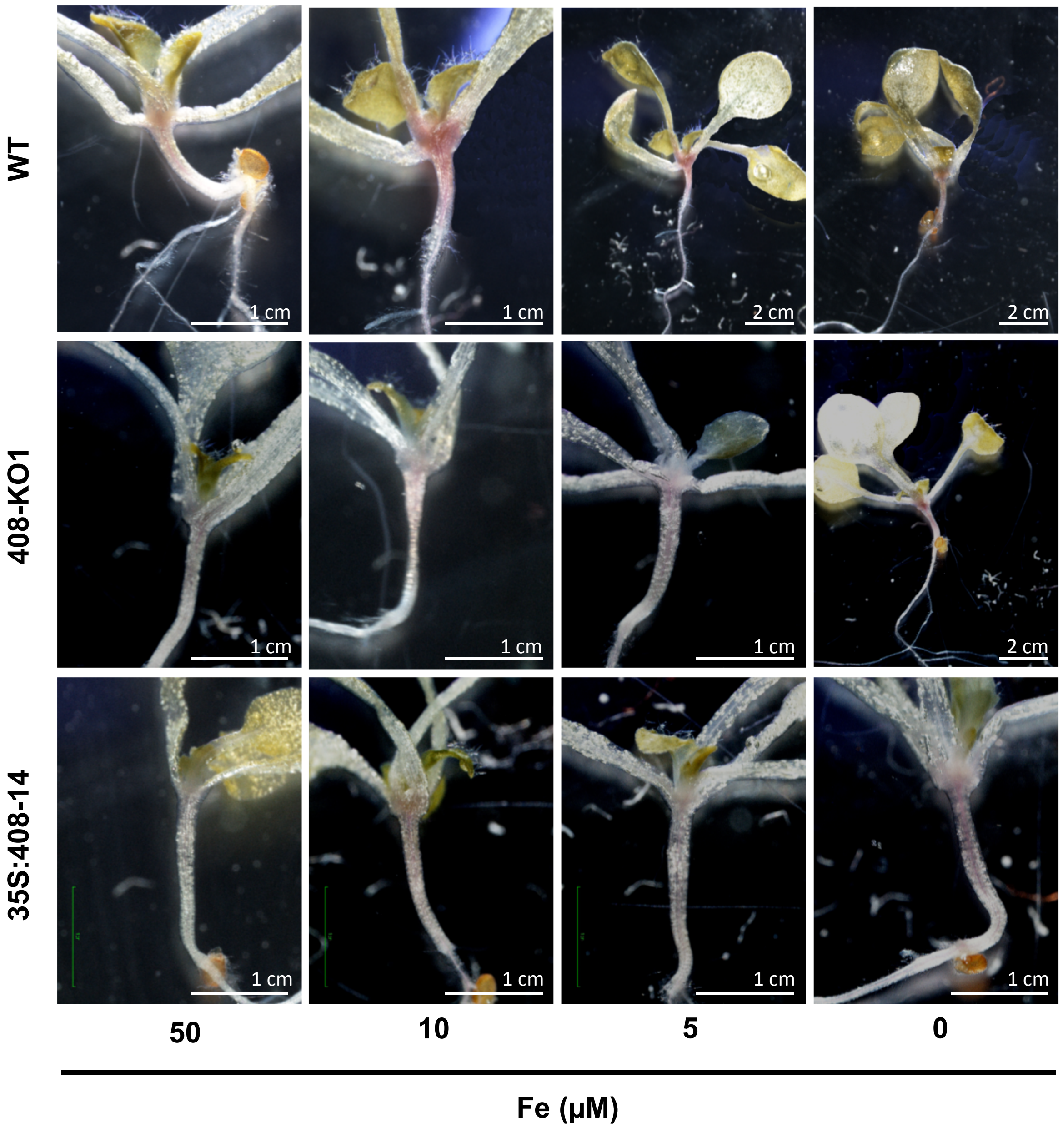

Figure S2. Lignin staining under iron deficiency conditions of altered *miR408* expression seedlings. Photographs of representative lignin stained stems from 15-day-old seedlings from wild-type (WT), 408-KO1 and 35S:408-14 lines grown on ½ MS plates containing 50, 5 and 0 µM Fe citrate. Seedlings were stained with phloroglucinol saturated with HCl and photographed using a microscope. Bars length indicate 1 or 2 cm as shown.

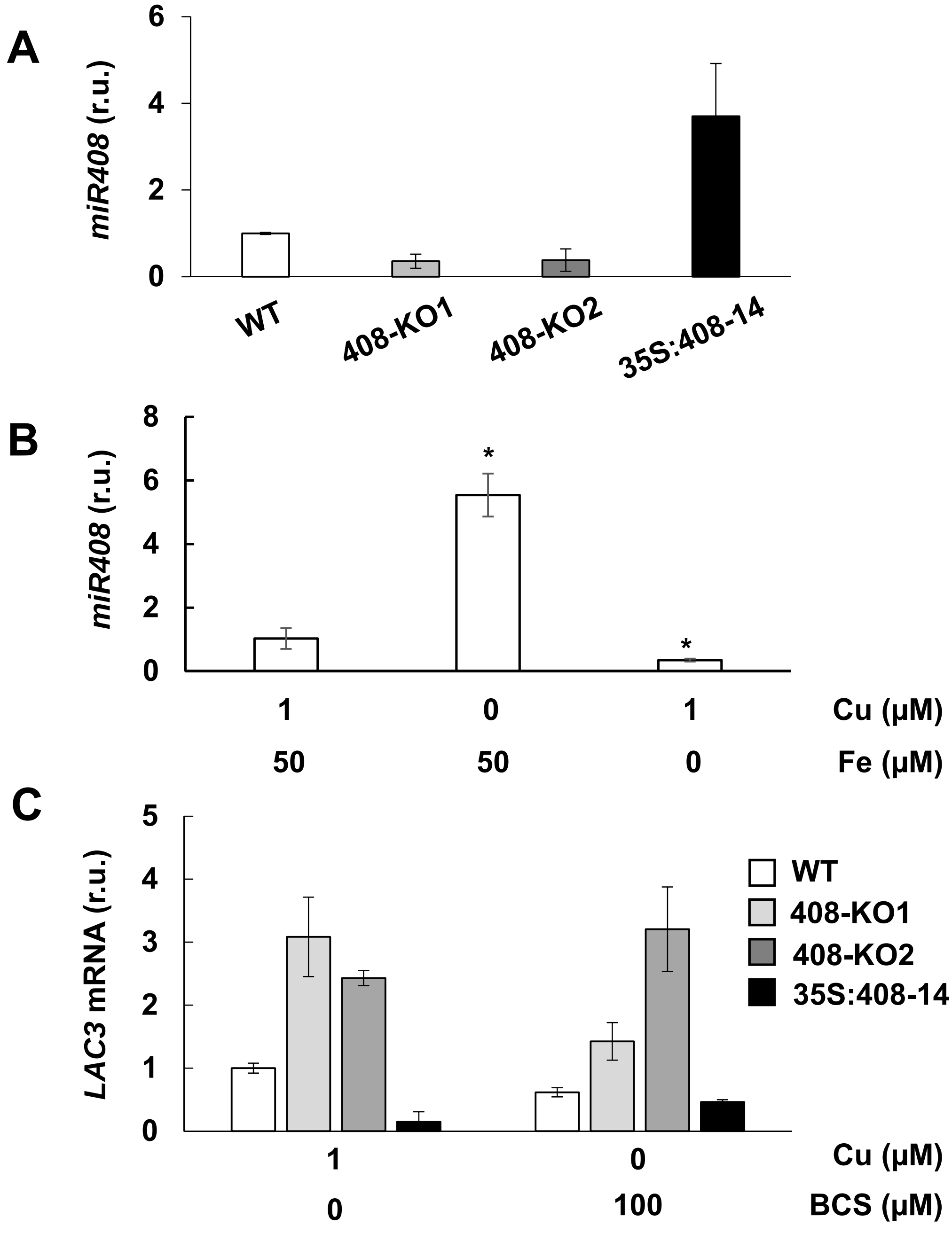

Figure S3. Expression pattern of *miR408* and *LAC3* genes under iron and copper deficiencies. **(A)** *miR408* expression in 7-day-old wild-type (WT), 408-KO1, 408-KO2 and 35S:408-14 seedlings grown in ½ MS medium under a pool of different conditions. **(B)** *miR408* expression in 7-day-old WT seedlings grown in media containing 50 and 0 µM Fe citrate and 1 or 0 µM $CuSO_4$. **(C)** *LAC3* expression in 7-day-old WT, 408-KO1, 408-KO2 and 35S:408-14 seedlings grown in ½ MS medium containing 1 µM $CuSO_4$ or 100 µM BCS. Total RNA was extracted and analyzed by RT-qPCR with specific oligonucleotides for *miR408* and *LAC3*. The relative expression in arbitrary units is represented. Values correspond to arithmetic means (2E-ΔΔCt) ± SD biological replicates. Asterisks indicate significant differences for the same group of samples respect to the WT line ($P < 0.05$) based on pair wise fixed reallocation randomization test.

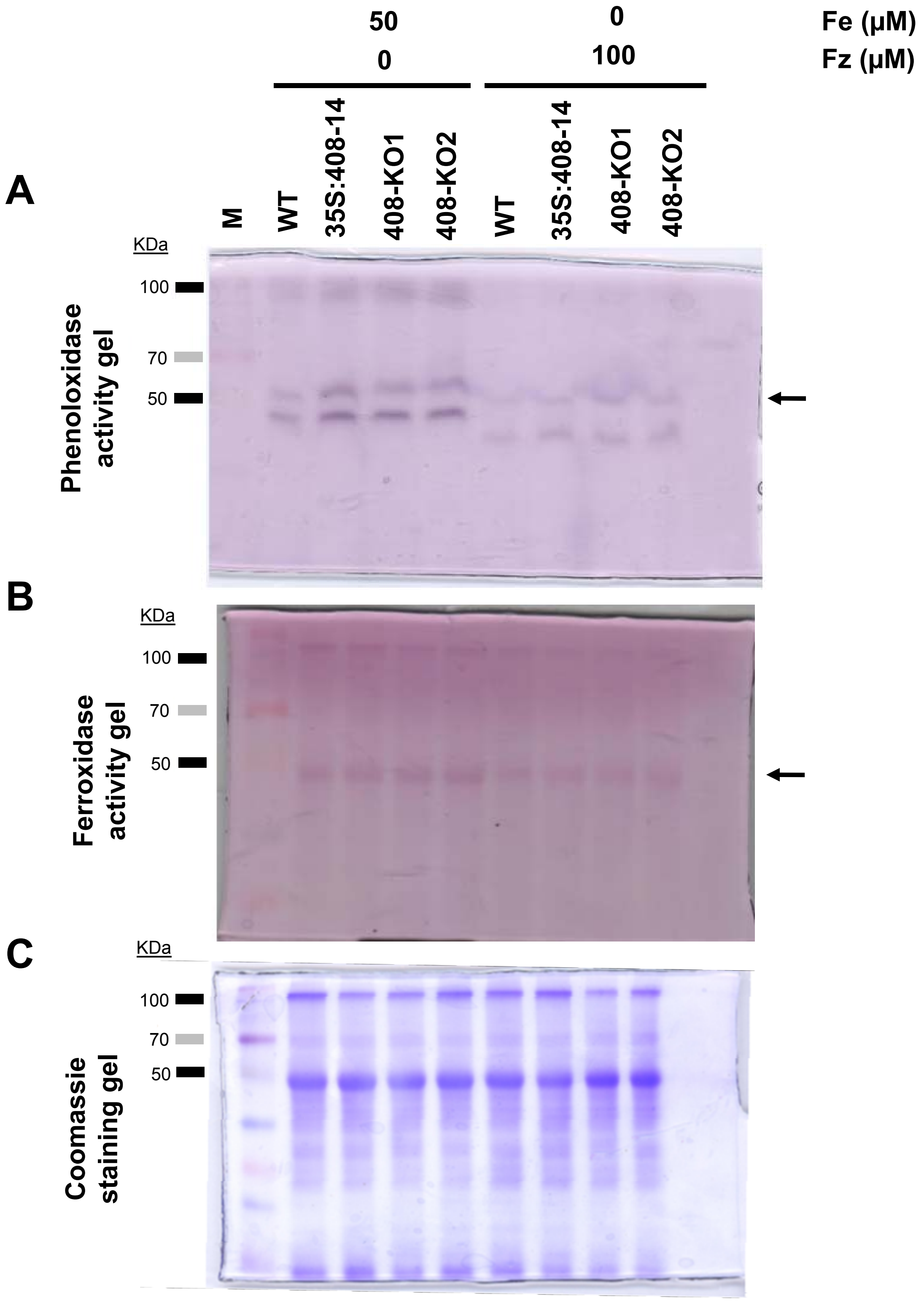

Figure S4. Phenoloxidase and ferroxidase activities in seedlings with altered *miR408* expression. A) Phenoloxidase, B) ferroxidase and C) Coomassie staining gels from activities shown in Figure 6. 7-day-old wild-type (WT), 408-KO1, 408-KO2 and 35S:408-14 seedlings, grown on ½ MS plates containing 50 µM Fe citrate and 100 mM ferrozine. A representative gel of at least 3 independent experiments is shown.

```
Saccharomyces_FET3  ------EI-----VEIVLNNQ-----DT-GTHPFHLHGHAFQTIQRDRTYDDALGEVPHSFDPDNHP
LAC1_AT1G18140      FGTKLFEVEFGSRLEIVFQGTSFLNIEN---HPLHVHGHNFFVVGRGFGNFDP--EKDP-KRYNLVD
LAC2_AT2G29130      RGTKVVVLKYKTTVELVLQGTSILGIEA---HPIHLHGFNFYVVGQGFGNFNP--ARDP-KHYNLVD
LAC3_AT2G30210      KGTKAYKLKYKSNVQIVLQDTSIVTPEN---HPMHLHGYQFYVVGSGFGNFNP--RTDP-ARFNLFD
LAC4_AT2G38080      TGTRLYKLPYNATVQLVLQDTGVIAPEN---HPVHLHGFNFFEVGRGLGNFNS--TKDP-KNFNLVD
LAC5_AT2G40370      RGTKLYKLKYGSRVQIVLQDTGIVTPEN---HPIHLHGYDFYIIAEGFGNFNP--KKDT-AKFNLED
LAC6_AT2G46570      NGTRAIVFEYGSRIQIIFQNTGTLTTEN---HPIHLHGHSFYVIGYGTGNYDQ----QT-AKFNLED
LAC7_AT3G09220      KSTSAKILKFNTTVEVVLQNHALIAAES---HPMHLHGFNFHVLAQGFGNYDP--SRDR-SKLNLVD
LAC8_AT5G01040      RKTSVKKIRFNSTVEIVLQNTAIISPES---HPMHLHGFNFYVLGYGFGNYDP--IRDA-RKLNLFN
LAC9_AT5G01050      RKTSVKTIRFNSTVEIVLQNTGILTPES---HPMHLHGFNFYVLGYGFGNYDP--IRDA-RKLNLFN
LAC10_AT5G01190     KATKLYKLPYNSTVQVVLQDTGNVAPEN---HPIHLHGFNFFVVGLGTGNYNS--KKDS-NKFNLVD
LAC11_AT5G03260     TGTRLSRVKFNTTIELVLQDTNLLTVES---HPFHLHGYNFFVVGTGVGNFDP--KKDP-AKFNLVD
LAC12_AT5G05390     KGTKLYKLKYGSRVQVVLQDTNIVTSEN---HPIHLHGYDFYIVGEGFGNFNP--KKDT-SKFNLVD
LAC13_AT5G07130     RGTKAYKLKFNSQVQIILQDTSIVTTEN---HPMHLHGYEFYVVGTGVGNFNP--NTDT-SSFNLID
LAC14_AT5G09360     FGTKVVVLDYNSSVELILQGTTVWASNI---HPIHLHGYNFYVVGSGFGNFDR--RKDP-LRYNLVD
LAC15_AT5G48100     LATEVKVIEFGQVVELVIQGTSLVGGGLD--HPMHLHGFSFYVVGVGFGNYNIS-EEDPSSRYNLYD
LAC16_AT5G58910     KGTKLYRLPYNATVQIVLQNTAMILSDN---HPFHLHGFNFFEVGRGLGNFNP--EKDP-KAFNLVD
LAC17_AT5G60020     NGTNLMVLPYNTSVELVMQDTSILGAES---HPLHLHGFNFFVVGQGFGNFDP--NKDP-RNFNLVD
```

Figure S5. Comparison of the copper binding and a putative ferroxidase signature motif of Fet3p and *Arabidopsis* laccases. Protein sequence alignment of the *Saccharomyces cerevisiae* multicopper oxidase Fet3p and the 17 postulated laccases from *Arabidopsis thaliana*. Conserved histidine residues involved in Cu binding are highlighted in yellow and a putative ferroxidase signature motifs D409 in Fet3p (Quintanar et al., 2007) in purple.

Table SI. Oligonucleotides used for real-time PCR.

Primer name	Sequence (5'-3')
ARPN F	TGACTCTCATGGCTGTGTCA
ARPN R	CACTACGTTGTGCATCCTCG
B-GLU23 F	CAATGAGCCATGGGTTTTCT
B-GLU23 R	TCCGGTAAGCTTCAACTGCT
bHLH39 F	CAGAGCTGCAAGAGCAAGTG
bHLH39 R	ACCAAGCCTAGTCGCAGAAA
CAT2 F	CGAGGTATGACCAGGTTCGT
CAT2 R	CTCCAGGCTCCTTGAAGTTG
CCR1 F	TATGTGGATGTTCGCGATGT
CCR1 R	GGTTCTTCTCGTCCTTGCAC
COPT2 F	CCTTTCGTATTTGGTGATGCT
COPT2 R	AAACACCTGCGTTAAAGGAC
CSD1 F	CAGCAGTGAGGGTGTTACG
CSD1 R	GCCCTGGAGACCAATGATG
F6'H1' F	TCTACACGTGCGTTCTCTGG
F6'H1' R	AAATCGGTTCCTCTCCGTTT
FIT F	TGTTCCCAATATTACTAA
FIT R	ATTTGAGTTTCTTCGCTT
FRO2 F	GTTGGTTTATAGCCCGACGA
FRO2 R	GGGCCGTAAGGACCTTCTAC
FRO3 F	GATTCTACTGGCTTCTCTTGG
FRO3 R	CTAATCCGGCCTTCACTAAC
FSD1 F	ACCGAAGACCAGATTACATA
FSD1 R	TGGCACTTACAGCTTCCCAA
IRT1 F	CCAACCAGACGGAAACATCT
IRT1 R	TACCAACTGCGTTCTTGCTG
LAC3 F	AACTGCTTTCACCAACCGTC
LAC3 R	TGGTAGCACGAAGGACATGT
LAC12 F	TCCTTTCCCTAAACCGGACC
LAC12 R	ACAGTCTCTTTGGTCGAGCA
LAC13 F	GAGCAGCACCAAACATCTCA
LAC13 R	TCTGCAACATGACAAGCACA
LAC17 F	GGTGGTCCCAATGTCTCTGA
LAC17 R	GCTTCAACAACCGTAACCGT
MCO3 F	AGTTTCCAGGGCCAACGATA
MCO3 R	TCCCGCCTTATCGACAATGA
miR408 F	CGCGAGCACAGAATTAATACGACTCACTATACGCGAGGGACCG
miR408 R	TGCAATGAAAGAAGACAAAGCG
UBQ10 F	CAGCGTCTCATCTTCGCTGGA
UBQ10 R	AAAACGAAGCGATGATAAAGAAG
